# Cyclin-Dependent Kinase 4 Phosphorylates and Positively Regulates PAX3-FOXO1 in Human Alveolar Rhabdomyosarcoma Cells

**DOI:** 10.1371/journal.pone.0058193

**Published:** 2013-02-28

**Authors:** Lingling Liu, Jing Wu, Su Sien Ong, Taosheng Chen

**Affiliations:** Department of Chemical Biology and Therapeutics, St. Jude Children's Research Hospital, Memphis, Tennessee, United States of America; The Institute of Cancer Research, United Kingdom

## Abstract

Alveolar rhabdomyosarcoma (ARMS) is an aggressive childhood muscle sarcoma with a 5-year survival rate of less than 30%. More than 80% of ARMSs harbor a PAX3-FOXO1 fusion transcription factor. However, expression of PAX3-FOXO1 in muscle cells alone is not sufficient and requires the loss of function of *Ink4a/ARF* to promote malignant proliferation of muscle cells *in vitro* or initiate ARMS tumor formation *in vivo*. This prompted us to examine the signaling pathways required to activate the function of PAX3-FOXO1 and to explore the functional interaction between the *Ink4a/ARF* and PAX3-FOXO1 signaling pathways. Here we report that inhibition of cyclin-dependent kinase 4 (Cdk4) by fascaplysin (a small molecule selective inhibitor of Cdk4/cyclin D1 that we identified in a screen for compounds that inhibit PAX3-FOXO1) led to inhibition of the transcriptional activity of PAX3-FOXO1 in ARMS cell line Rh30. Consistent with this finding, activation of Cdk4 enhanced the activity of PAX3-FOXO1. *In vitro* kinase assays revealed that Cdk4 directly phosphorylated PAX3-FOXO1 at Ser^430^. Whereas fascaplysin did not affect the protein level of PAX3-FOXO1, it did increase the cytoplasmic level of PAX3-FOXO1 in a portion of cells. Our findings indicate that Cdk4 phosphorylates and positively regulates PAX3-FOXO1 and suggest that inhibition of Cdk4 activity should be explored as a promising avenue for developing therapy for ARMS.

## Introduction

Rhabdomyosarcoma (RMS) is the most common soft tissue sarcoma in children. Histopathologically, two subtypes of RMS have been identified, embryonal (ERMS) and alveolar (ARMS), each with distinct clinical and genetic characteristics. Most of the more aggressive ARMSs are associated with a 2;13 chromosomal translocation, generating a PAX3-FOXO1 fusion product―a cytogenetic hallmark of ARMS. PAX3-FOXO1 is associated with a poor prognosis and a 5-year survival rate of less than 30% for ARMS patients, and once metastasis occurs, ARMS becomes resistant to conventional chemotherapy and radiotherapy. Therefore, understanding the regulation of PAX3-FOXO1 to develop new therapeutic agents is urgently needed [1], [2]. The unique expression, function, and subcellular localization of PAX3-FOXO1 contribute to its oncogenic behavior by modifying cell growth, differentiation, and migration [2]. However, elucidating the oncogenic function of PAX3-FOXO1 has been challenging, partly due to conflicting data generated under different cellular contexts. Whereas early studies using avian and rodent cell lines showed that PAX3-FOXO1 acted as an oncogene that caused cell transformation, later studies by ectopically expressing PAX3-FOXO1 in various murine and human ERMS cell lines suggested that PAX3-FOXO1 could either stimulate or inhibit cell proliferation and apoptosis [3]. While the underlying mechanism was unclear, these conflicting observations indicated that the function of PAX3-FOXO1 might be highly dependent on the cellular environment. In a recent study using primary human skeletal muscle cells, a cell type relevant to RMS, Linardic et al. [4] showed that expression of PAX3-FOXO1, accompanied by the a loss of expression of tumor suppressor p16^INK4A^, could promote these cells to bypass the senescence growth arrest checkpoint and proliferate inappropriately. In other studies, Keller at al. [5], [6] showed that ARMS occurred at a low frequency in mice with a conditional *Pax3-foxo1* knock-in. High frequencies of ARMS tumor formation occurred only in mice with *Pax3-foxo1* knock-in accompanied by a conditional *Trp53* or *INK4a/ARF* loss of function, suggesting that expression of PAX3-FOXO1 is necessary but not sufficient to induce ARMS at high frequencies. These observations also implied that the activity of PAX3-FOXO1 requires activation of another signaling pathway, which is possibly mediated by the loss of *INK4a/ARF* function.

To identify the cellular signaling pathways that contribute to regulating the function of PAX3-FOXO1, we sought a cell-based screening approach that would identify compounds that affect PAX3-FOXO1 transcriptional activity. By screening a library of kinase inhibitors, we identified fascaplysin, a selective inhibitor of cyclin-dependent kinase 4 (Cdk4)/cyclin D1, that inhibits PAX3-FOXO1 transcriptional activity. Consistent with this observation, we found that activation of Cdk4 led to enhanced activity of PAX3-FOXO1. We also found that Cdk4 directly phosphorylated PAX3-FOXO1 and that inhibition of PAX3-FOXO1 by fascaplysin partially retained PAX3-FOXO1 in the cytoplasm. Our primary aim was to identify cellular pathways that regulate the function of PAX3-FOXO1. We identified such a pathway in which Cdk4 phosphorylates to positively regulate the activity of PAX3-FOXO1.

## Materials and Methods

### Cell Culture

Rh30, Rh41, RD, NIH3T3, JR-1 cells, and Rh30_PRS9 (Rh30 stably transfected with a PAX3-FOXO1–responsive firefly luciferase reporter [pGL4.20-6XPRS9, or 6 X PRS9, which contains both the paired domain and homeodomain recognition sites]) have been described previously [7]–[9]. All cells were cultured in an incubator with a humidified atmosphere maintained at 5% CO_2_ and 95% air at 37°C. Cells were split every 3 days at 90% to 95% confluency. Phenol red–free DMEM (Invitrogen, Carlsbad, CA) was used for all luminescence assays.

### Plasmids

Full length or partial (1–423 and 1–459) PAX3-FOXO1 coding sequence from pEGFP-PAX3-FOXO1 (GFP-PF) [8] was subcloned into pGEX-5X-1 glutathione S-transferase (GST) expression vector (GE Healthcare, Piscataway, NJ) to create GST-PAX3-FOXO1 (GST-PF), GST-PF (1–423), and GST-PF (1–459). Mutant constructs (serine 430 to alanine or aspartic acid) were generated by using a QuickChange site-directed mutagenesis kit (Stratagene, La Jolla, CA) and appropriate mutated primers. pCMV-Cdk4 and pCMV-Cdk4DN were previously described by Dr. Sander van den Heuvel [10] and obtained from Addgene (Cambridge, MA). pCMV-cyclin D1 was described by Dr. Yue Xiong [11] and was also obtained from Addgene.

### PAX3-FOXO1 Transactivation Assay

NIH3T3 cells were co-transfected with pEGFP-C3 or pEGFP-PAX3-FOXO1 (wild-type or mutants), with or without Cdk4 or cyclin D1, 6 X PRS9 firefly luciferase reporters (pGL4.20-6 X PRS9) [8], and a constitutively expressed Renilla luciferase reporter (PRL-TK, or TK-renilla, used as a control for transfection efficiency) (Promega, Madison, WI) by using FuGENE 6 according to the manufacturer’s instructions. Then, 20 h after transfection, 10,000 cells were plated in each well of a 96-well culture plate and grown for an additional 4 h before luciferase activity was measured using the Dual-Glo Luciferase Assay System (Promega) according to the manufacturer’s instructions. The firefly luciferase activity was normalized to that of Renilla luciferase as previously described [7].

### Kinase Inhibitor Screening

**Figure 1:**
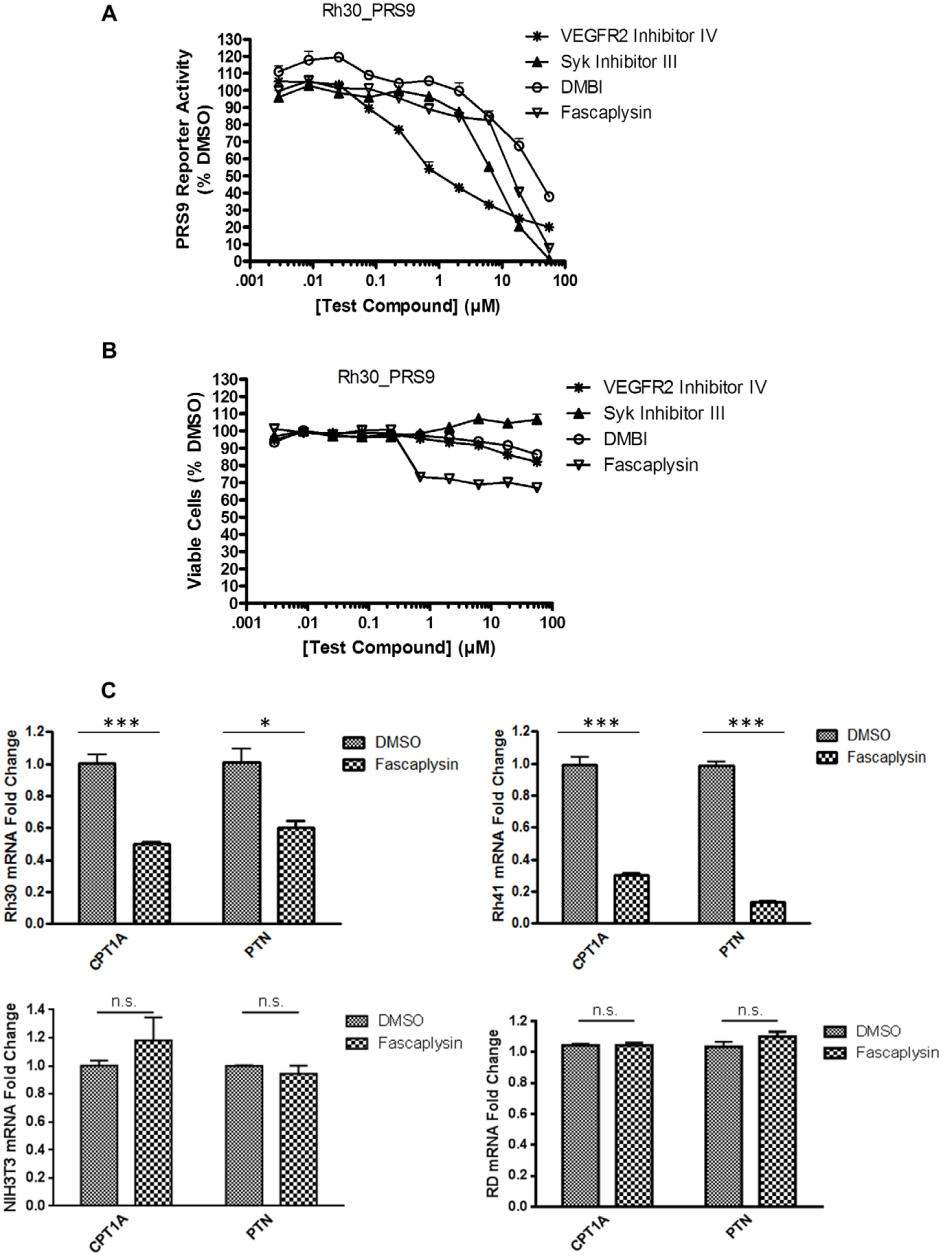
Identification of kinase inhibitors that inhibit PAX3-FOXO1 transcriptional activity. Rh30_PRS9 cells were treated with increasing concentrations (2.8 nM to 56 µM) of compounds for 2 h before determination of **A**) PRS9 reporter activity and **B**) viable cells (percentage relative to DMSO-treated cells). **C**) Fascaplysin decreased the expression of CPT1A and PTN in a PAX3-FOXO1-dependent manner. Rh30, Rh41, NIH3T3, and RD cells were treated with 1 µM fascaplysin or DMSO for 2 h. CPT1A or PTN mRNA levels in samples treated with fascaplysin were normalized to that of cells treated with DMSO (samples treated with DMSO were arbitrarily set as 1). * *p*<0.05; *** *p*<0.001; ns *p*>0.05.

Rh30_PRS9 were seeded at 5,000 cells per well in 25 µl of medium into solid white 384-well tissue culture-treated plates 24 h before compound treatment. Then, 140 nL of compound at either 1 mM or 10 mM was added to each well (final compound concentration was 5.6 µM or 56 µM; final DMSO concentration was 0.56%), and the plates were incubated for 2 h before a luciferase reporter assay using the SteadyLiteHTS Luciferase Assay System (PerkinElmer, Waltham, MA) and cell viability assay using the CellTiter-Glo Luminescent Cell Viability Assay (Promega) as described previously [8], [12]. The compounds used for the screen contained 160 protein kinase inhibitors from the InhibitorSelect 384-Well Protein Kinase Inhibitor Library I (EMD Millipore, Billerica, MA). Data are expressed as percentage of reporter activity of firefly luciferase or percentage of viable cells in the presence of compound compared with DMSO vehicle control (set as 100%) as previously described [8]. At 5.6 µM, 2 compounds [AGL 2043 (CAS 226717-28-8, a PDGFR inhibitor) and VEGFR2 inhibitor IV (CAS 216661-57-3)], and at 56 µM, 5 additional compounds [Syk inhibitor III (CAS 1485-00-3), DMBI (CAS 5812-07-7, a PDGFR inhibitor), fascaplysin (CAS 114719-57-2, a selective Cdk4/cyclin D1 inhibitor), Chelerythrine Chloride (CAS 3895-92-9, a PKC inhibitor), and GSK-3β inhibitor I] showed >50% of inhibition in luciferase reporter activity and the difference in inhibition of luciferase reporter activity and inhibition of viability was greater than 30%. Inhibitors of PKC and GSK-3β have previously been reported to inhibit the transcriptional activity of PAX3- FOXO1 [1], [8]. For dose response analysis, compounds were first diluted in DMSO (1∶3 serial dilutions from 10 mM; final compound concentrations ranged from 2.8 nM to 56 µM) and added to cells as described above. Four compounds significantly inhibited the luciferase activity in a dose-dependent manner but only slightly decreased cell viability (the difference in maximal inhibition of luciferase reporter activity and the maximal inhibition of viability was greater than 30%) are shown in Figure 1.

### Real-time Reverse Transcription–polymerase Chain Reaction

Real-time reverse transcription–polymerase chain reaction (RT-PCR) was used to measure the levels of mRNA. Extraction of total RNA from cellular lysates; preparation of cDNA; primers for PAX3-FOXO1, carnitine palmitoyltransferase 1A (CPT1A), pleiotrophin (PTN), and glucose-6-phosphate dehydrogenase (GAPDH); and the conditions for PCR have been described previously [7], [8], [13]. Real-time PCR was performed using an iCycler iQ real-time PCR detection system (Bio-Rad, Hercules, CA). The mRNA levels were quantified as previously described [7]. Briefly, the cycle threshold (Ct) values of each gene of interest and of GAPDH were calculated for each sample, and then the normalized value was derived by subtracting the Ct value of GAPDH from that of the gene of interest (ΔCt). Data are shown as mRNA fold change (2^–ΔΔCt^) relative to the mRNA level of the corresponding transcript in the control samples as indicated.

### Purification of GST Tagged PAX3-FOXO1

Various versions of GST-tagged PAX3-FOXO1 (PF), GST-PF, GST-PF (1–423), GST-PF (1–459), and GST-PF (1–459) S430A were expressed in *Escherichia coli* BL21 for purification. Briefly, 100 mL of bacterial cultures was spun down, and pellets were resuspended in 10 mL of bacterial lysis buffer (1 mM EDTA, 0.35 M NaCl, 200 mM PMSF, 50 mg/ml lysozyme, 500 mM DTT, and protease inhibitor cocktail in PBS) at 4°C for 30 min. The lysate was sonicated and centrifuged, and the supernatant was then incubated with 100 µL of GST beads for 2 h at 4°C, and the beads were washed three times with wash buffer (50 mM Tris-HCl, pH 8.0, 0.5 M NaCl, 0.1 mM EDTA, 0.1 mM EGTA, 0.5% Triton X-100, 0.5% Tween-20, and 5 mM DTT). Beads were then either mixed with SDS loading buffer for Western blotting or resuspended in 1 × kinase buffer (Cell Signaling, Danvers, MA) for *in vitro* kinase assay.

### Cell Transfection and Protein Pull-down Assays

Rh30 cells were transfected with either pEGFP-C3 vector or pEGFP-PAX3-FOXO1 for 24 h, harvested, and lysed as previously described [7]. Chromotek-GFP trap resin (Allele Biotechnology, San Diego, CA) was added to the lysates and incubated at 4°C for 2 h. Beads were then washed three times with PBS-T (0.1% Tween) and either mixed with SDS loading buffer for Western blotting or resuspended in with 1 X kinase buffer for *in vitro* kinase assay.

### Western Blot Analysis

Western blot analysis was performed as previously described [7]. Anti-FOXO1 antibodies (H-128; sc-11350) and anti-GFP antibodies (sc-9996) were obtained from Santa Cruz Biotechnology (Santa Cruz, CA); anti-actin antibodies (A5441) were obtained from Sigma (St. Louis, MO); anti-Cdk4 antibody (#2906), anti-cyclin D1 antibody (#2926), anti-GST antibody (#2624), and phospho-(Ser) Cdks substrate antibody (#2324), which recognizes the phosphor-serine in a KS*P motif, were obtained from Cell Signaling (Danvers, MA).

### *In vitro* Cdk4 Kinase Assay

In the *in vitro* kinase assays, 100 ng of Cdk4/cyclin D1 (SignalChem, Richmond, Canada) was used per reaction. Kinase assays were performed as described before [8], [12] in 25 µL reactions with ∼1 µg (in 5 µL beads) of substrate protein, 5 µM cold ATP, and 5 µCi of [γ-^32^P] ATP (6000 Ci/mmol) (PerkinElmer, Santa Clara, California). The reactions were incubated at 30°C for 60 min and then loaded to SDS-PAGE. To visualize the amount of the samples used, the gel was stained using Coomassie Brilliant Blue R-250 (Bio-Rad, Hercules, CA). The stained gel was then desiccated by using a gel dryer (Bio-Rad). The dried gel was then subjected to overnight exposure in the Storage Phosphor Screen (GE Healthcare, Pittsburgh, PA). Phosphor images were obtained using the Storm scanner (GE Healthcare). [γ-^32^P] ATP was omitted in samples prepared to be used with the phospho-(Ser) Cdks substrate antibody.

### Microscopy

Rh30 or NIH3T3 cells were transfected with pEGFP-PAX3-FOXO1 (wild-type or S430A mutant) for 24 h and treated with 1 µM fascaplysin or DMSO for 30 min. Cells were then washed with PBS, fixed with 4% paraformaldehyde (Electron Microscopy Sciences, Hatfield, PA), and washed again with PBS and water, and mounted with Vectashield mounting medium with DAPI (Vector, Burlingame, CA) for imaging analysis. Cell images were taken using an Olympus IX-51 or a Zeiss LSM 510 NLO Meta, and analyzed using the cell analysis tool (Cellomics vHCS Scan NucTrans Bioapplication) within the Thermo Fisher (Pittsburgh, PA) Cellomics vHCS Toolbox [14], and the ratio of the average intensity of the GFP signals in the nucleus and cytoplasm was determined. Mann-Whitney non-parametric analysis was performed using GraphPad Prism (Graphpad Software, La Jolla, CA).

### Statistical Analysis

In all figures, results are expressed as the mean ± standard deviation (SD) of at least 3 independent experiments, and error bars indicate SD. Student’s *t*-test was used to determine the statistical significance of the difference between paired samples. Differences were considered significant if *p*<0.05 (*), 0.01 (**) or 0.001 (***) and non-significant (ns) if *p*>0.05. Where applicable, sample pairs are noted with a line in the figure.

## Results

### Cdk4 Inhibitor Inhibits PAX3-FOXO1 Transcriptional Activity

To identify compounds that inhibit the activity of PAX3-FOXO1, we screened an ARMS cell line Rh30_PRS9 (Rh30 stably expressing PAX3-FOXO1 responsive reporter 6 X PRS9) against a collection of 160 kinase inhibitors [8]. To minimize the effect of non-specific cytotoxic compounds, we chose a shorter compound treatment time (2 h) for our screen and identified four compounds that significantly inhibited PAX3-FOXO1 transcriptional activity in a dose-dependent manner (Fig. 1A) but only slightly decreased cell viability (less than 30% inhibition) (Fig. 1B): VEGFR2 inhibitor IV (CAS 216661-57-3), Syk inhibitor III (CAS 1485-00-3), DMBI (CAS 5812-07-7, a PDGFR inhibitor), and fascaplysin (CAS 114719-57-2, a selective Cdk4/cyclin D1 inhibitor) [15].

Our study focused on fascaplysin, because Cdk4 may play an important role in regulating the function of PAX3-FOXO1, suggested by previous studies showing that a loss of *Ink4a/ARF*, which leads to activation of Cdk4, promoted PAX3-FOXO1’s oncogenic function [4]–[6]. To confirm the effect of fascaplysin on inhibiting the transcriptional activity of PAX3-FOXO1, we showed that the mRNA levels of two PAX3-FOXO1 transcriptional targets, CPT1A [7] and PTN [13], were reduced by 50% and 40%, respectively, after 2 h of treatment with fascaplysin in Rh30 cells. The inhibitory effect of fascaplysin on the mRNA levels of CPT1A and PTN was also observed in another ARMS cell line Rh41, but not in PAX3-FOXO1 negative cell lines NIH3T3 and RD (Fig. 1C).

### Cdk4 Phosphorylates PAX3-FOXO1

**Figure 2:**
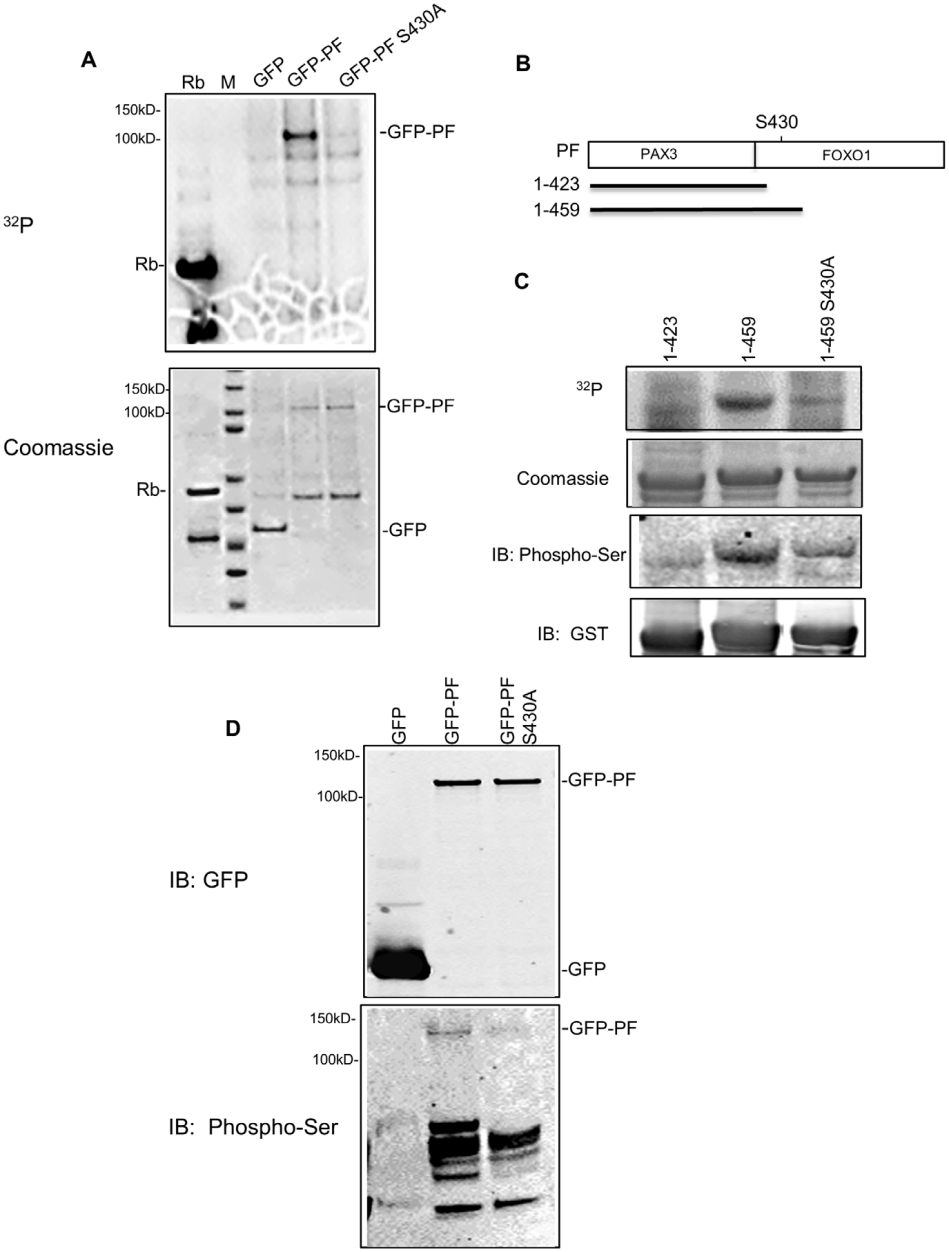
Cdk4 phosphorylates PAX3-FOXO1 at Ser^430^ *in vitro* and *in vivo*. **A**) *In vitro* kinase assay using GFP or GFP-PAX3-FOXO1 (GFP-PF; wild-type or S430A mutant) pulled down from transfected Rh30 cells. Top panel: ^32^P Phospho-image. Bottom panel: protein substrates revealed by Coomassie blue staining. **B**) Schematic diagram of three GST-PF fusion proteins (showing only the PAX3-FOXO1 portion). PF, full length PAX3-FOXO1; 1–423, the N-terminal 423 residues of PAX3-FOXO1; 1–459, N-terminal 459 residues of PAX3-FOXO1; S430, Serine 430; PAX3, the PAX3 portion of PAX3-FOXO1; FOXO1, the FOXO1 portion of PAX3-FOXO1. **C**) *In vitro* kinase assay using bacterially expressed PAX3-FOXO1. From top to bottom panel: ^32^P Phospho-image (^32^P); protein substrates revealed by Coomassie blue staining (Coomassie); Western blot using phosphor-ser CDKs substrate antibody (IB: Phospho-Ser); and Western blot using anti-GST monoclonal antibody (IB: GST). **D**) Detection of phosphorylation using GFP or GFP-PF (wild-type or S430A mutant) proteins pulled down from transfected Rh30 cells. Top panel: Western blot using anti-GFP monoclonal antibody; Bottom panel: Western blot using phosphor-ser CDKs substrate antibody. Rb, positive control for Cdk4 activity; M, protein marker indicating molecular weight (kD).

Phosphorylation of PAX3-FOXO1 by protein kinases has been shown to regulate its transcriptional activity [1], [8]. We hypothesized that the activities of PAX3-FOXO1 would be regulated by its functional interactions with Cdk4 and sought to determine whether Cdk4 could phosphorylate the PAX3-FOXO1 protein. In an *in vitro* kinase assay, we found that purified Cdk4/cyclin D1 phosphorylates GFP-PAX3-FOXO1, but not GFP, both expressed and purified from Rh30 cells (Fig. 2A). Rb protein, a known substrate of Cdk4, was used as a positive control for Cdk4 activity. These findings indicate that Cdk4 directly phosphorylates PAX3-FOXO1 protein *in vitro*.

Cdks often recognize and phosphorylate a serine or threonine that is followed by a proline, and it was reported that Ser^249^ of FOXO1 (in K^248^S^249^P^250^) is a residue preferentially phosphorylated by Cdks [16], [17]. PAX3-FOXO1 contains the C-terminal portion of FOXO1 (amino acids 211 to 655), and Ser^249^ in FOXO1 corresponds to Ser^430^ in PAX3-FOXO1. To determine whether Ser^430^ of PAX3-FOXO1 (in K^429^S^430^P^431^) is phosphorylated by Cdk4, we made a GFP-PAX3-FOXO1 S430A mutant, and showed that the mutation significantly reduced the phosphorylation by Cdk4/cyclin D1 (Fig. 2A). Furthermore, we generated three fragments of GST-PAX3-FOXO1: GST-PAX3-FOXO1 (1–423), GST-PAX3-FOXO1 (1–459), and GST-PAX3-FOXO1 (1–459)S430A (Fig. 2B). In an *in vitro* kinase assay, whereas 1–459, the fragment that contains Ser^430^, was significantly phosphorylated by Cdk4/cyclin D1, phosphorylation of the fragment with either Ser^430^ deleted (1–423) or mutated (1–459S430A) was significantly decreased (Fig. 2C, top panel). Similar results were obtained when a phospho-(Ser) Cdks substrate antibody, which recognizes phosphor-serine in a KS*P motif, was used in samples from kinase reactions in which [γ-^32^P] ATP was omitted (Fig. 2C, third panel from top). These findings suggest that Cdk4 phosphorylates the Ser^430^ residue of PAX3-FOXO1 *in vitro*.

To determine whether PAX3-FOXO1 is phosphorylated at Ser^430^ *in vivo*, we expressed various GFP-PAX3-FOXO1 constructs in Rh30 cells and pulled down the proteins to examine their phosphorylation status by using the phospho-(Ser) Cdks substrate antibody. As shown in Fig. 2D (bottom panel), the phosphor-specific antibody recognized the wild-type GFP-PAX3-FOXO1 (GFP-PF); phosphorylation decreased in GFP-PAX3-FOXO1 with a Ser^430^ to Ala^430^ mutation (GFP-PFS430A). These results indicate that PAX3-FOXO1 is phosphorylated at Ser^430^ *in vivo*. Taken together, our findings show that Cdk4 phosphorylates PAX3-FOXO1, and Ser^430^ is one of the phosphorylation sites.

### Activation of Cdk4 Enhances PAX3-FOXO1 Transcriptional Activity

**Figure 3:**
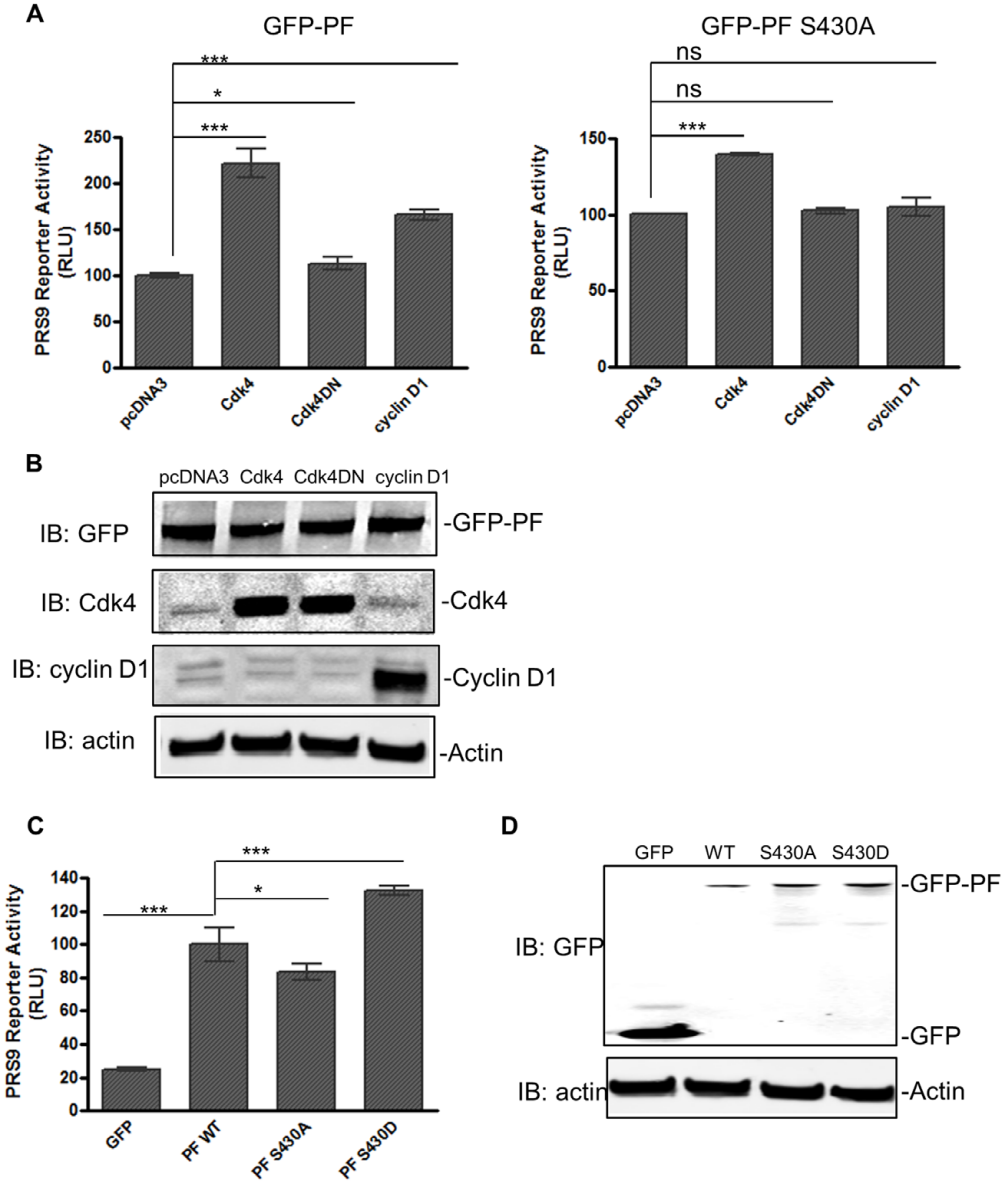
Activation of Cdk4 enhances PAX3-FOXO1-mediated gene expression. **A**) NIH3T3 cells were co-transfected with wild-type GFP-PAX3-FOXO1 (left panel) or GFP-PAX3-FOXO1 S430A (right panel), 6 X PRS9 reporter, TK-renilla (PRL-TK), and one of the plasmids as indicated. Comparisons were made between other samples and a sample transfected with pcDNA3 (set as 100 relative luciferase units, or RLU). Cdk4, pCMV-Cdk4 (wild-type); Cdk4DN, pCMV-Cdk4DN (inactive Cdk4); cyclin D1, pCMV-cyclin D1. **B**) Protein expression in A) was confirmed by Western blot analysis using anti-GFP antibody (IB: GFP), anti-Cdk4 antibody (IB: Cdk4), anti-cycin D1 (IB: cyclin D1), and anti-actin (IB: actin). Actin was used as a loading control. GFP-PF, GFP-PAX3-FOXO1. **C**) NIH3T3 cells were transfected with 6 X PRS9 reporter, TK-renilla, and one of the plasmids indicated. PF WT, wild-type GFP-PAX3-FOXO1; PFS430A, GFP-PAX3-FOXO1S430A; PFS430D, GFP-PAX3-FOXO1S430D. Comparisons were made between other samples and a sample transfected with wild-type GFP-PAX3-FOXO1 (set as 100 RLUs). **D**) Protein expression in B) was confirmed by Western blot analysis. Actin was used as a loading control. Luciferase activity was measured 24 h after transfection. Firefly luciferase activity was normalized using Renilla luciferase activity. * *p*<0.05; *** *p*<0.001.

Since PAX3-FOXO1 is phosphorylated by Cdk4/cyclin D1, and the selective inhibitor of Cdk4/cyclin D1 fascaplysin inhibited the transcriptional activity of PAX3-FOXO1, it is most likely that activation of Cdk4 will lead to the activation of PAX3-FOXO1. To test this hypothesis, we transfected PAX3-FOXO1 with or without co-transfection of Cdk4 or cyclin D1 into NIH3T3 cells, a cell line commonly used in studying PAX3-FOXO1 activity. Overexpression of a wild-type Cdk4 increased PAX3-FOXO1 transcriptional activity by more than 2-fold over that of an empty vector control (pcDNA3) (Fig. 3A). Overexpression of cyclin D1, which regulates the activity of Cdk4, also enhanced the activity of PAX3-FOXO1. However, an inactive kinase-dead Cdk4 (Cdk4DN) only marginally affected the activity of PAX3-FOXO1. The activation effect of Cdk4 on PAX3-FOXO1 was significantly reduced (to less than 50%) when a S430A mutation was introduced. Fig. 3B shows the expression levels of all constructs and indicates that the level of PAX3-FOXO1 was similar under all four transfection combinations. These data indicate that activation of Cdk4 leads to enhanced activity of PAX3-FOXO1. To provide further evidence that the activity of PAX3-FOXO1 is regulated by Cdk4 through phosphorylation, we tested the activity of a phosphorylation-deficient mutant (GFP-PAX3-FOXO1 S430A), in which the mutation of serine to alanine places a hydrophobic side chain at position 430 and renders it deficient of phosphorylation, and a phosphomimetic mutant (GFP-PAX3-FOXO1 S430D) in which serine was mutated to a negatively charged aspartate to mimic phosphorylation. As shown in Fig. 3C, the S430A mutant was 17% less active, whereas the S430D mutant was 32% more active than the wild-type PAX3-FOXO1. These findings, together with the results shown in Fig. 2, suggest that Cdk4 phosphorylates PAX3-FOXO1 to promote its transcriptional activity, and Ser^430^ is one of the phosphorylation sites.

### Fascaplysin Enhances the Cytoplasmic Localization of PAX3-FOXO1

**Figure 4:**
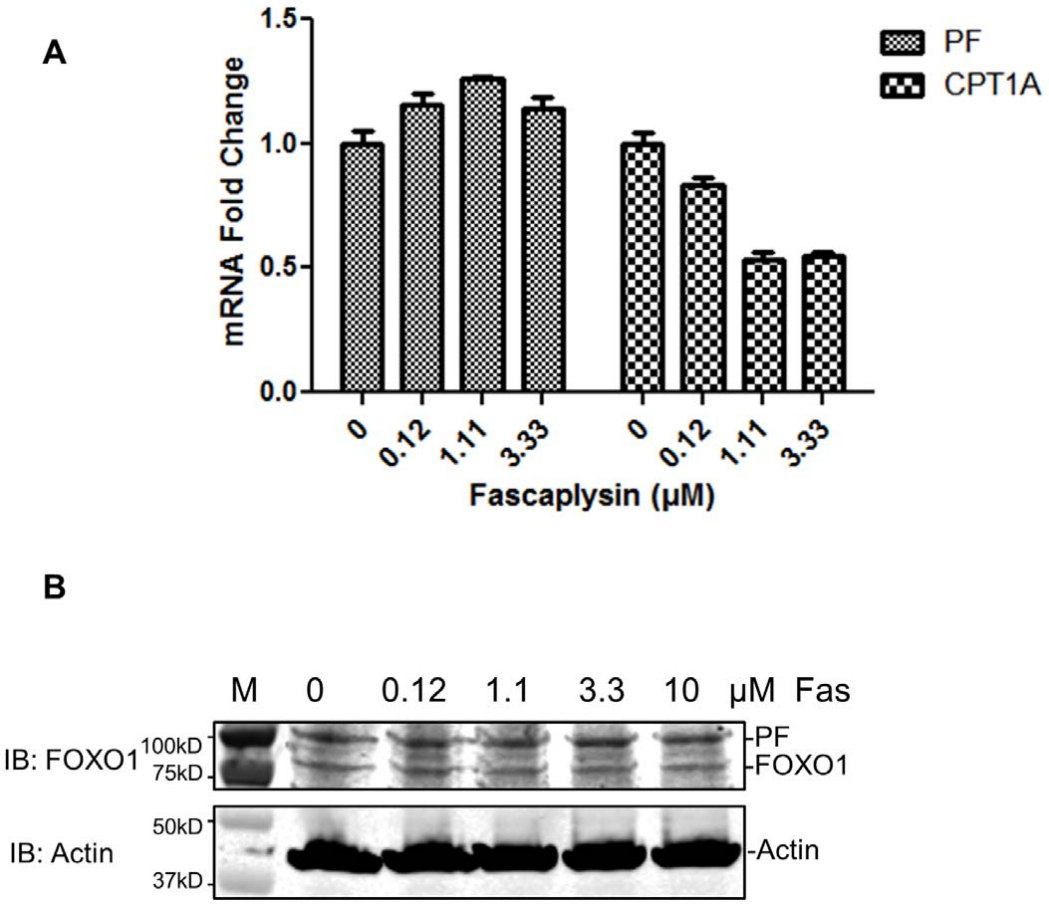
Fascaplysin treatment does not affect the levels of PAX3-FOXO1 in Rh30 cells. **A**) Fascaplysin reduced the mRNA levels of CPT1A but not PAX3-FOXO1 (PF) in Rh30 cells. **B**) Fascaplysin did not affect protein levels of PAX3-FOXO1. Top panel: Western blot using anti-FOXO1 antibody. Bottom panel: Western blot using anti-actin antibody. Cells were treated with indicated concentrations of fascaplysin (Fas) for 2 h. M, protein molecular weight marker.

To determine the mechanism by which fascaplysin inhibits the transcriptional activity of PAX3-FOXO1, we first analyzed the mRNA and protein levels of PAX3-FOXO1 in response to 2 h of fascaplysin treatment in Rh30 cells. As shown in Fig. 4, fascaplysin decreased the transcriptional activity of PAX3-FOXO1, as evidenced by the decreased mRNA levels of CPT1A, without decreasing the mRNA (Fig. 4A) or protein (Fig. 4B) levels of PAX3-FOXO1, suggesting that some mechanism other than decreased protein levels is responsible for the inhibitory effect of fascaplysin on PAX3-FOXO1.

**Figure 5:**
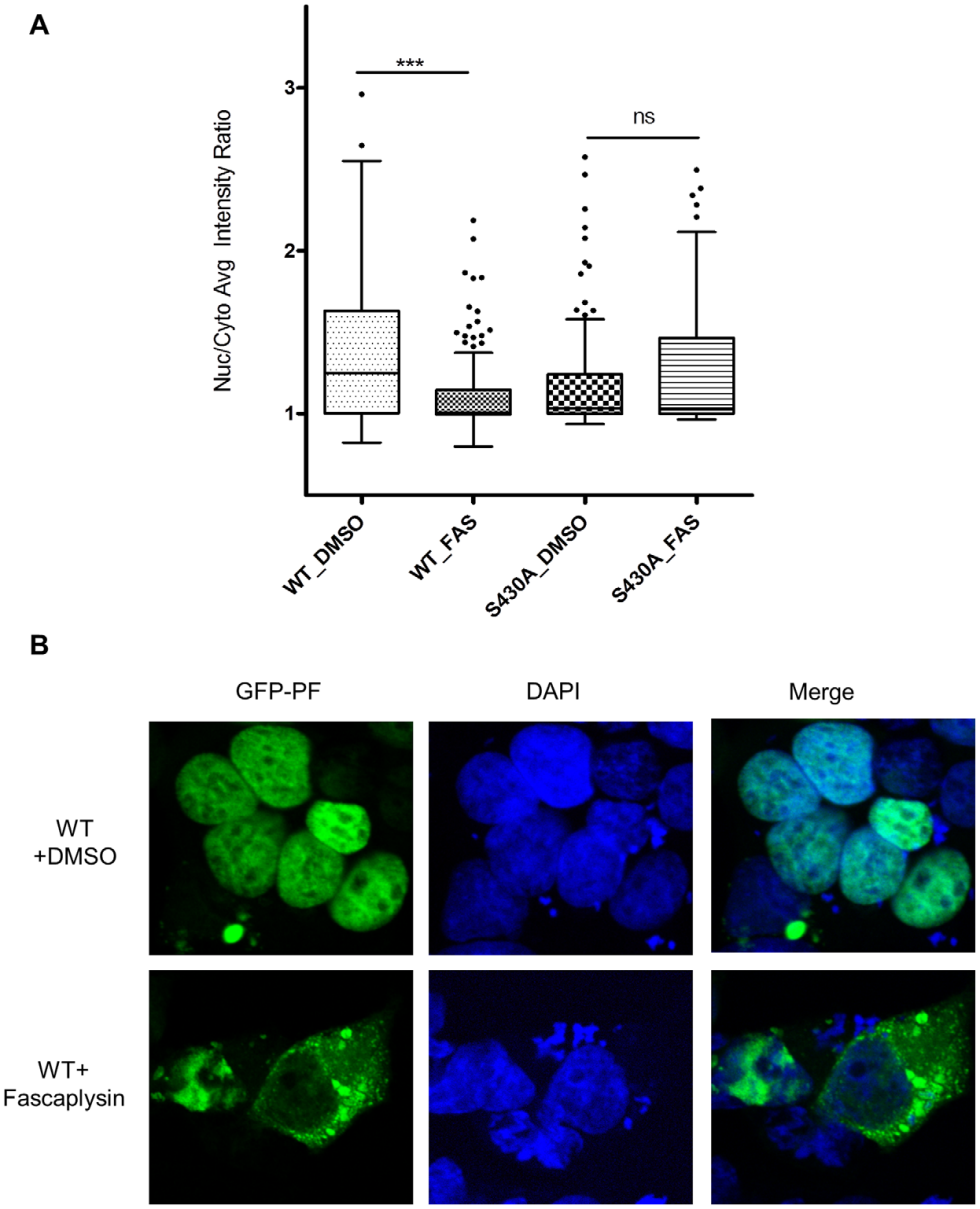
Fascaplysin enhances PAX3-FOXO1 cytoplasmic localization in Rh30 cells. **A**) Scatter plot of GFP-PAX3-FOXO1 subcellular localization. The ratio of the average intensities of the GFP signals in the nucleus and cytoplasm was determined as described in Materials and Methods. Each dot in the plot represents one cell. Cells were transfected with wild-type (WT) GFP-PAX3-FOXO1 (GFP-PF), or GFP-PF S430A mutant (S430A) for 24 h, followed by treatment with either DMSO or 1 µM fascaplysin (FAS) for 30 min. Cells were then fixed and stained with DAPI. **B**) Representative images.

The subcellular localization of a transcription factor is critical for its transcriptional activity and is well-studied for FOXO1 but not for PAX3-FOXO1 [14], [16]–[18]. Whereas activation of AKT signaling retains FOXO1 in the cytoplasm, PAX3-FOXO1 is insensitive to AKT activity and is predominantly nuclear-localized, suggesting that the regulation of subcellular localization by phosphorylation is different between FOXO1 and PAX3-FOXO1 [18]. Recently, Cdks was reported to phosphorylate Ser^249^ of FOXO1 and resulted in its cytoplasmic localization [16], [17]. Since Cdk4 phosphorylates Ser^430^ in PAX3-FOXO1 (corresponds to Ser^249^ in FOXO1), we tested whether inhibition of Cdk4 by fascaplysin alters the subcellular localization of PAX3-FOXO1. PAX3-FOXO1 is known to predominantly nuclear-localized [18]. When a cell analysis tool (Cellomics vHCS Scan NucTrans Bioapplication) [14] was used to analyze the images and determine the ratio of the average intensities of the GFP signals in the nucleus and cytoplasm, wild-type GFP-PAX3-FOXO1 is more nuclear localized than GFP-PAX3-FOXO1 S430A, and treatment with fascaplysin for 30 min significantly enhanced the cytoplasmic localization of wild-type GFP-PAX3-FOXO1 but not the phosphorylation-resistant mutant S430A (Fig. 5A). Representative images were shown in Fig. 5B to illustrate the fascaplysin-mediated cytoplasmic localization of PAX3-FOXO1 in a portion of the cells. The effect of fascaplysin in promoting cytoplasmic localization of PAX3-FOXO1 was also observed in NIH3T3 cells (Fig. S1). These findings suggest that Cdk4 enhances the transcriptional activity of PAX3-FOXO1 by phosphorylating and possibly either inhibiting the cytoplasmic translocation or promoting the nuclear translocation of PAX3-FOXO1 in a fascaplysin-sensitive manner.

### Fascaplysin Exerts Inhibitory Effect on ARMS Cells

**Figure 6:**
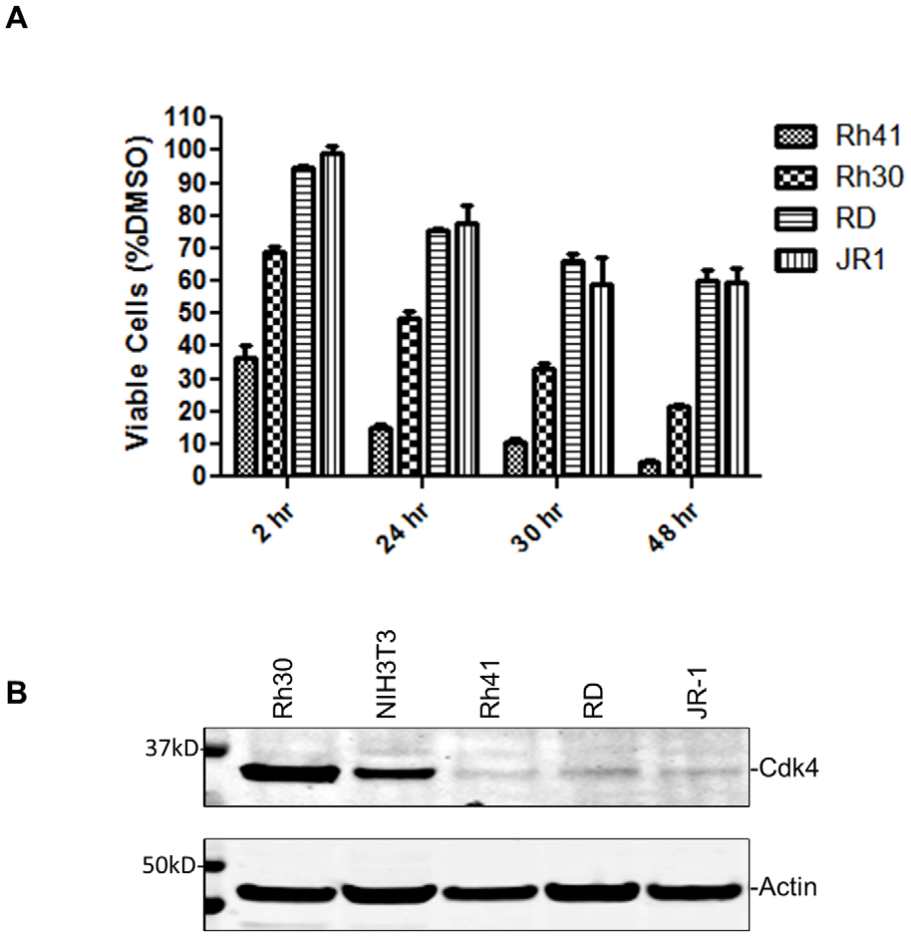
Fascaplysin inhibits growth of ARMS cells. **A**) ARMS cells (Rh30 and Rh41) and ERMS cells (RD and JR1) were treated with 0.69 µM fascaplysin for indicated times before cell viability was determined. Fascaplysin at 0.69 µM started to inhibit cell viability of Rh30 cells as shown in Figure 1B. **B**) Cdk4 levels in various cell lines.

Downregulating PAX3-FOXO1 by using RNA interference (RNAi) markedly decreased anchorage-independent growth and cell motility of Rh30 cells [7], [13]. Although fascaplysin is toxic to both ARMS (Rh41 and Rh30; PAX3-FOXO1–positive) and ERMS cells (RD and JR-1; PAX3-FOXO1–negative), ARMS cells were more sensitive to fascaplysin than ERMS cells in a cell viability assay (Fig. 6A). All cell lines tested expressed Cdk4 (Fig. 6B), which is in agreement with previously reported results [19]. Interestingly, Rh41, in which the level of endogenous PAX3-FOXO1 is much higher than in Rh30 [20], was most sensitive to fascaplysin. The level of Cdk4 is not highest in Rh41, suggesting that the sensitivity to fascaplysin might be determined by the levels of both Cdk4 and PAX3-FOXO1 in ARMS cells, and the inhibitory effect of fascaplysin on ARMS cells is PAX3-FOXO1–dependent.

## Discussion

Cdk4 directly phosphorylates and enhances the function of PAX3-FOXO1. We identified Ser^430^ as a residue phosphorylated by Cdk4; however, Ser^430^ might not be the only residue phosphorylated by Cdk4, since mutation of Ser^430^ to Ala reduced, but did not abolish, the phosphorylation of PAX3-FOXO1 by Cdk4. Phosphorylations of PAX3-FOXO1 at various residues have been shown to regulate its transcriptional activity [1], [8], [21], [22]. Dietz et al identified phosphorylation of PAX3-FOXO1 at serine 201 [21] and serine 205 [22], consistent with the results reported by Amstutz et al in which they provided evidence that phosphorylation in this region of PAX3-FOXO1 influences its transcriptional activity [1].

We found that treatment with fascaplysin did not change the protein level but enhanced the cytoplasmic localization of PAX3-FOXO1, suggesting that Cdk4 phosphorylates PAX3-FOXO1 and possibly either inhibits its translocation from the nucleus to the cytoplasm or promotes its translocation from the cytoplasm to the nucleus, thereby enhancing its transcriptional activity. The modest effect of fascaplysin on the localization of PAX3-FOXO1 suggests that changing in its cellular localization presumably only partially contributes to the regulation of PAX3-FOXO1 by CDK4. As expected, the phosphorylation-deficient S430A became less sensitive to fascaplysin. Although both the wild-type PAX3-FOXO1 and the S430A mutant are predominantly nuclear-localized, the wild-type is more nuclear localized than the S430A mutant. Cdks often recognize and phosphorylate the motif of serine (S) or threonine (T) followed by proline (P), with a preferred consensus motif being (S/T)PX(R/K), where X is a polar amino acid such as aspartic acid. Although S430 is the only residue that exists in a preferred consensus motif, there are 5 threonines and 10 serines that are followed by a proline in the PAX3-FOXO1 sequence. Regulation of protein trafficking is a complex process; comprehensive identification of all residues phosphorylated by Cdk4 and investigation of their functional significance will help elucidate the role of Cdk4-mediated phosphorylation in regulating the subcellular localization of PAX3-FOXO1.

The previous discovery that the loss of p16^INK4A^ function enabled the oncogenic effect of PAX3-FOXO1 [4]–[6] implied that the activity of PAX3-FOXO1 requires the activation of another pathway, possibly mediated by the loss of *INK4a/ARF* function. Activation of the Cdk4 pathway is one of the downstream effects of the loss of *INK4a/ARF* function. The identification of Cdk4 as a pathway that positively regulates the function of PAX3-FOXO1 suggests that the activation of Cdk4 caused by the loss of *INK4a/ARF* function might be responsible for promoting the oncogenic function of PAX3-FOXO1. Indeed, decreased p16^INK4a^ levels were observed in ARMS cell lines that are PAX3-FOXO1–positive but not in ERMS cell lines that are PAX3-FOXO1–negative, further indicating the functionally significant association between PAX3-FOXO1 expression and the loss of p16^INK4a^ function in promoting ARMS tumor formation [4].

A previous study showed that Cdk4 was expressed in 73% of ARMS and ERMS tumors. A Cdk4/Cdk6 inhibitor, PD0332991, was able to cause G1 arrest of RMS cells *in vitro* and delayed the growth of RMS xenografts *in vivo* [19], suggesting that pharmacologic inhibition of Cdk4/Cdk6 is a therapeutic strategy for RMS. The observation that a decreased p16^INK4a^ level, which leads to increased activation of Cdk4, correlates with PAX3-FOXO1 expression in ARMS tumor formation [4] would predict that pharmacologic inhibition of Cdk4 is effective for treating ARMS. Indeed, we observed that fascaplysin reduced cell viability more effectively in ARMS than in ERMS cells. Although fascaplysin is a toxic compound whose cytotoxic effect is not specific to RMS [23], our observation suggests that it is feasible to develop pharmacologic inhibitors of Cdk4 as therapeutics for ARMS.

In summary, our study showed that Cdk4 phosphorylates and activates PAX3-FOXO1, thereby promoting its oncogenic function. Pharmacologic inhibition of Cdk4 is therefore a valuable and feasible approach to developing treatment for ARMS.

## Supporting Information

Figure S1 **Fascaplysin enhances PAX3-FOXO1 cytoplasmic localization in NIH3T3 cells. A)** and **B)** Cells were transfected and processed as described in Figure 5.
(TIF)
