# Supplementary figures and images for: Cyclin-Dependent Kinase 4 Phosphorylates and Positively Regulates PAX3-FOXO1 in Human Alveolar Rhabdomyosarcoma Cells
Source: PLoS One. 2013 Feb 28;8(2):e58193. doi: 10.1371/journal.pone.0058193 (PMC3585270; doi:10.1371/journal.pone.0058193)

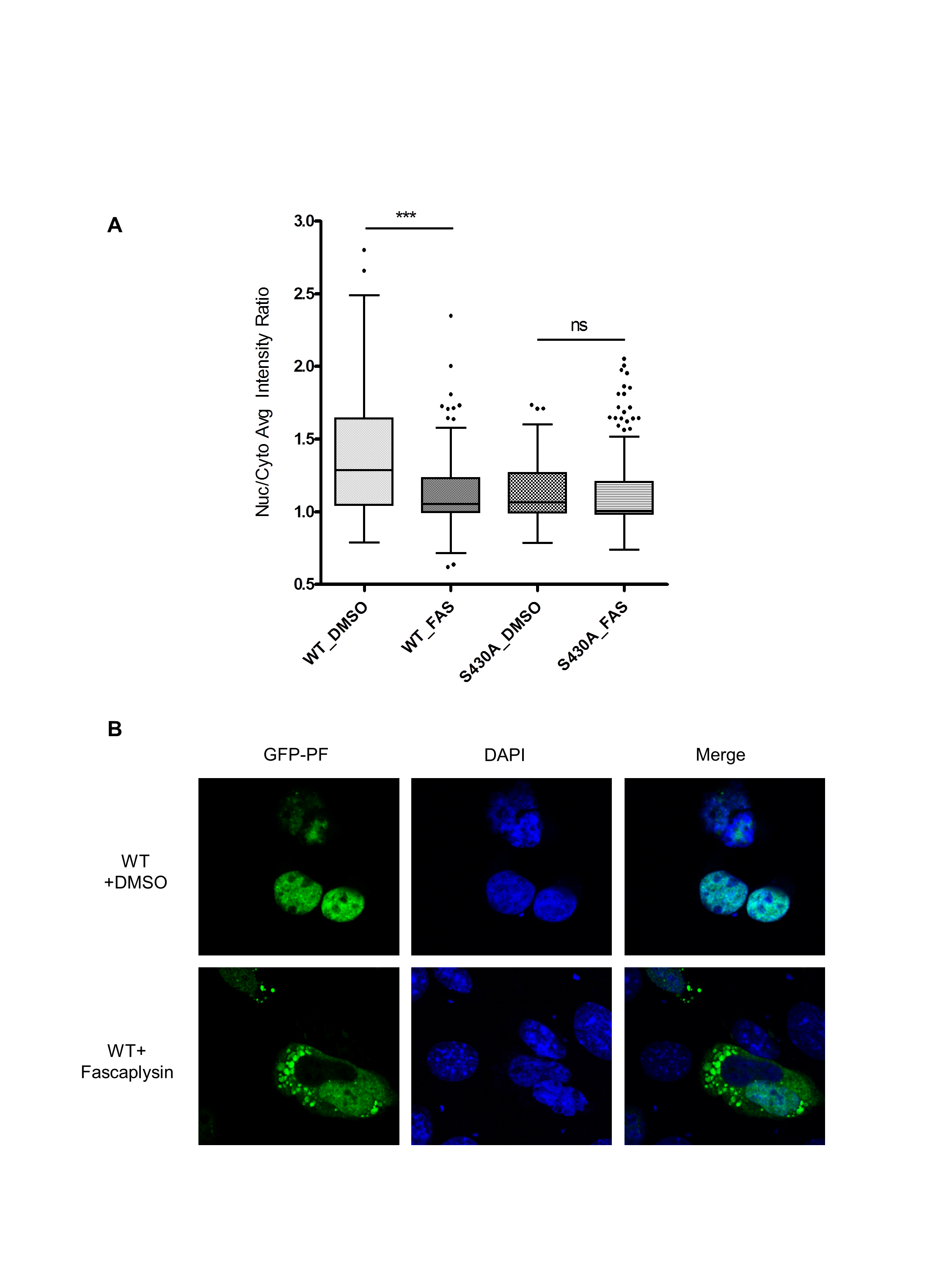

Supplement: Figure S1 — Fascaplysin enhances PAX3-FOXO1 cytoplasmic localization in NIH3T3 cells. A) and B) Cells were transfected and processed as described in Figure 5. (TIF) [file pone.0058193.s001.tif]
